# Open-Source 3D-Printable Optics Equipment

**DOI:** 10.1371/journal.pone.0059840

**Published:** 2013-03-27

**Authors:** Chenlong Zhang, Nicholas C. Anzalone, Rodrigo P. Faria, Joshua M. Pearce

**Affiliations:** 1 Michigan Tech Open Sustainability Technology Laboratory, Michigan Technological University, Houghton, Michigan, United States of America; 2 Department of Materials Science and Engineering, Michigan Technological University, Houghton, Michigan, United States of America; 3 Department of Electrical and Computer Engineering, Michigan Technological University, Houghton, Michigan, United States of America; UMR-S665, INSERM, Université Paris Diderot, INTS, France

## Abstract

Just as the power of the open-source design paradigm has driven down the cost of software to the point that it is accessible to most people, the rise of open-source hardware is poised to drive down the cost of doing experimental science to expand access to everyone. To assist in this aim, this paper introduces a library of open-source 3-D-printable optics components. This library operates as a flexible, low-cost public-domain tool set for developing both research and teaching optics hardware. First, the use of parametric open-source designs using an open-source computer aided design package is described to customize the optics hardware for any application. Second, details are provided on the use of open-source 3-D printers (additive layer manufacturing) to fabricate the primary mechanical components, which are then combined to construct complex optics-related devices. Third, the use of the open-source electronics prototyping platform are illustrated as control for optical experimental apparatuses. This study demonstrates an open-source optical library, which significantly reduces the costs associated with much optical equipment, while also enabling relatively easily adapted customizable designs. The cost reductions in general are over 97%, with some components representing only 1% of the current commercial investment for optical products of similar function. The results of this study make its clear that this method of scientific hardware development enables a much broader audience to participate in optical experimentation both as research and teaching platforms than previous proprietary methods.

## Introduction

As much of the Internet now relies on free and open-source software (FOSS), open source is becoming a standard method of software development [1], [2]. Open source developmental methods are a fundamentally new, decentralized, participatory and transparent system to create software in contrast to the closed box, top-down, and secretive standard commercial approach [3]. FOSS provides an alternative to expensive and proprietary systems, reduces research and development costs, and also showcases alternatives to the linear hierarchical structure used to design technology and products. Furthermore, FOSS has demonstrated the efficiency of collaboration, demand-driven innovation and the power of the Internet to provide for a global collective good. The use of FOSS software has assisted a number of engineering fields to do tasks such as CAD, EDA, CFD, FEA, computer vision and even project management. In the sciences FOSS has driven down the costs of numerical simulation in a number of fields ranging from psychotherapy [4], neural circuit reconstruction [5], and genomic sequences annotation [6]. Due to this tremendous success of FOSS development, the concept has spread outside of software to areas such as education [7], [8], appropriate technology for sustainable development [9], [10], science [11], nanotechnology [12], [13] and medicine [14], [15]. Both academic and non-academic scientists are accustomed to this line of thinking as both historical knowledge sharing and the Internet enabled new era of networked science have demonstrated the power of working together [16], [17]. Today, the rise of open-source hardware is poised to drive down the cost of doing experimental science and put state-of-the-art scientific tools in the hands of everyone [18].

To assist in this aim, this article introduces a library of open-source 3-D-printable optics equipment, which can be used as a flexible, low-cost public-domain tool set for developing both research and teaching optics hardware. First, the use of parametric open-source designs using an open-source computer aided design package is described to customize the optics hardware for any application. Second, details are provided to use open-source 3-D printers (additive layer manufacturing) to fabricate the primary components and construct complex, multi-component optics-related devices. Third, the use of the open-source electronic prototyping platforms are illustrated as control devices for optics experiments. Overall, this paper describes open-source optics development, system requirements, features, advantages, and known limitations; and discuss future directions for this method of scientific hardware development to radically reduce the cost of research and science education.

## Materials and Methods

### OpenSCAD

The optics designs were developed in OpenSCAD [19]. OpenSCAD is an open-source, script-based computer aided design application. It is used by writing code to describe the geometric specifications of the required object by using three primitive shapes (cylinder, sphere and cube) and complex polygons using polygon, polyline and the 2D–3D extrusion commands. OpenSCAD allows for parametric designs, which is the ability to alter a design to specifications by changing the parameters of the geometry of an object. This allows changes to be made to the design easily and quickly by simply changing the value of user-defined variables.

**Figure 1:**
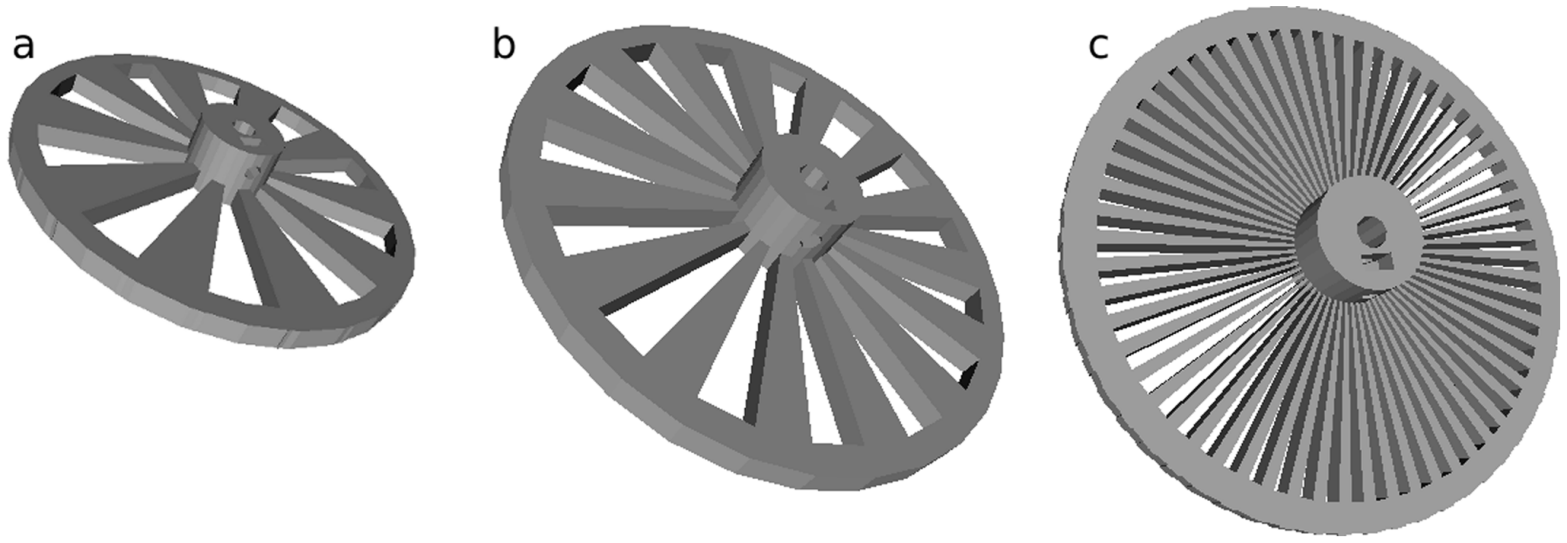
Rendered parametric design in OpenSCAD on an open-source optical chopper wheel with a) 10 slots, b) 15 slots and c) 60 slots.

To illustrate the utility of parametric design consider an optical chopper wheel, which modulates the frequency of a light beam. For a given chopper wheel setup the target frequency can be adjusted by the number of slots in the chopper wheel. Normally, this involves the separate purchase of a new wheel for each experiment, and the experimenter does not always know beforehand what the optimal operational frequency is for a given application. A parametric design from OpenSCAD is shown in Figure 1a, b, and c, where the slot number for an open source optical chopper wheel has been adjusted by changing a single variable in the code from 10, 15 and 60 slots providing ranges of chopper frequency of 20 Hz-1 kHz, 30 Hz-1.5 kHz, and 120 Hz-6 kHz, respectively [20]. OpenSCAD directly exports the geometry to an STL file (STereoLithography), which is used for 3-D printer open-source slicing programs (e.g. Skeinforge [21] or Slic3r [22]), which is in turn transformed to g-code, which provides the vectors for tool path (3-D printer extruder head).

### Open-source 3-D Printing and Microcontrollers

**Figure 2:**
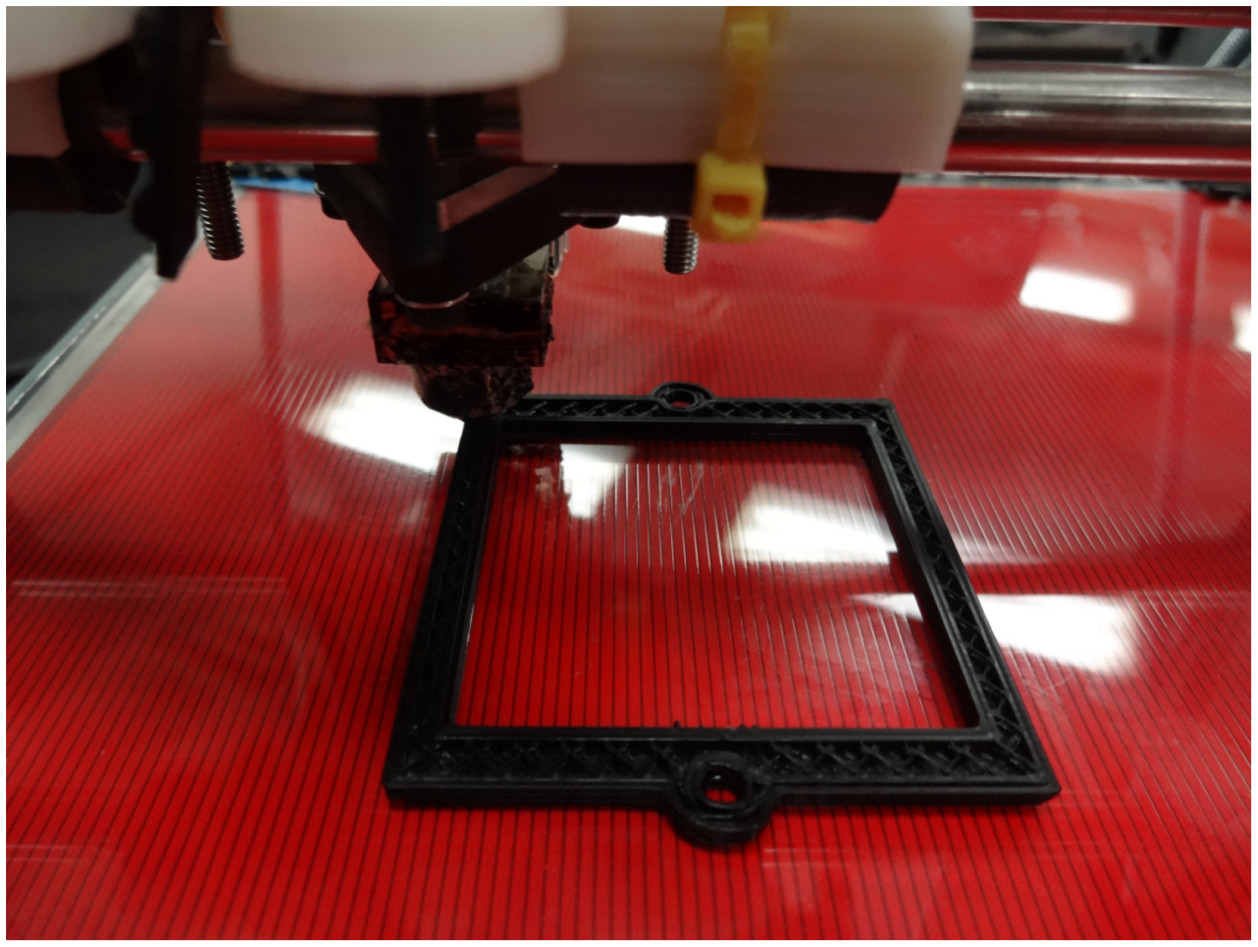
An open-source self-replicating rapid prototyper printing a 3-D optical component – filter bracket.

Recently, the development of open-source 3-D printers like the RepRap [23], which cost less than $1000 [24], have made the cost of rapid prototyping accessible to most university laboratories [18]. RepRap’s open-source and self-replicating nature (approximately 50% of its own parts can be self-printed) makes it an extremely useful platform for open-source fabrication. The printing process for the additive layer manufacture of optical experimental components is a sequential layer deposition. The RepRap extruder intakes a filament of the working material (acrylonitrile-butadiene-styrene [ABS] and Polylactic acid **[**PLA] polymers), heats it, and extrudes it through a nozzle where deposits a 2-D layer of the working material, then the Z (vertical) axis will raise, and the extruder will deposit another layer on top of the first. In this way it can build three dimensional models from a series of two dimensional layers [25]. Figure 2 shows the RepRap printing out a component of a filter wheel system [26]. These models can be customized for a given optical setup using OpenSCAD and printed. The current versions of the RepRap are controlled by an Arduino microcontroller, which is prototyping platform based on the ATMEL ATmega328, a high-performance, low-power AVR 8-Bit microcontroller. Arduino microcontrollers are a family of open-source, low-cost integrated circuits that contains a core processor, memory and analog and digital I/O (input/output) peripherals [27]. As Arduino microcontrollers are relatively easy to use they have been applied in a vast number of science and engineering areas including tools such as holographic microscope [28], portable system for high-speed multispectral optical imaging [29], and an LED stimulator system for vision research [30]. Here the automation capabilities of the Arduino can be combined with the rapid prototyping of the RepRap to make sophisticated customized equipment such as an automated filter wheel changer.

Using the combination of OpenSCAD, RepRap 3-D printing and Arduino automation a multi-component open-source optics library has been designed, built and is described below.

## Results

### Open-Source Optics Library

**Figure 3:**
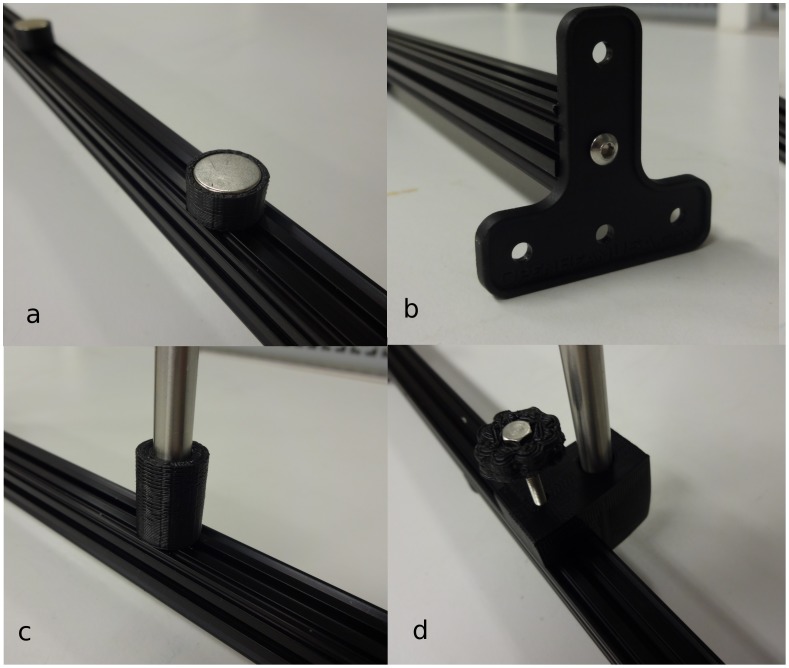
Open-source optical rail fabricated from OpenBeam using a printed a) magnetic base or b) T-brackets, c) simple rod holder, and d) off-set rod holder.

**Figure 4:**
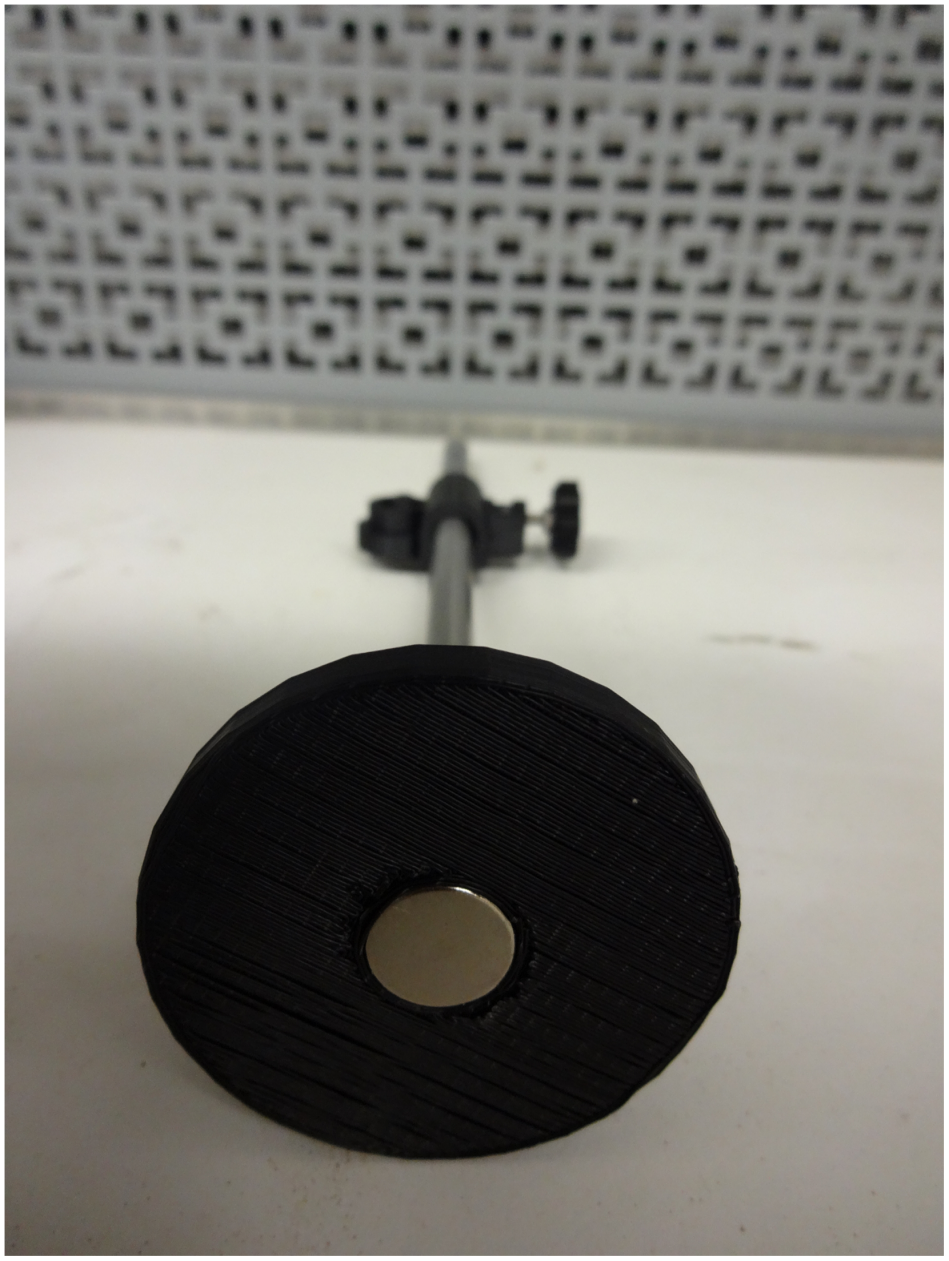
Magnetic optics base.

The open-source optics library is built from standard low-cost parts available in most hardware stores and customizable printed parts. First an optical rail is fabricated from an open-source aluminum extrusion system called OpenBeam as seen in Figure 3. An optical rail is a long, straight, sturdy rail onto which optical components such as light sources and lenses can be bolted down and easily shifted along the length of the rail. Commercial optical rail sells for around $380/m ($115/ft), while OpenBeam is available from $10–12/m. The OpenBeam system is an open-sourced, miniaturized t-slot construction system that utilizes standard metric (m3) nuts and bolts to connect to an extruded 1 m aluminum rod. The OpenBeam is converted into an optical rail using a printable magnetic base (Figure 3a) [31], which holds the beam securely on a steel table or desk. For those not using metal tables, two OpenBeam T-brackets can either be purchased or printed to mount to any non-metallic surface (Figure 3b) [32]. Optical components are attached via 8 mm diameter smooth rods centered directly above the beam using a simple rod holder (Figure 3c) [33] or via an off-set rod holder (Figure 3d) [34]. While avoiding the substantial cost of an optical table, some optical experimental setups with a non-linear experimental optical setup can be fabricated using a magnetic optics base [35] as seen in Figure 4, which has a small cylinder opening in the bottom meant to glue in a magnet. The optics base quasi-permanently holds the position of the optical component on a magnetic surface with more flexibility than a standard optical table although not as much stability. It must be pointed out here that all of the components shown are parametric in the OpenSCAD design. So, for example, other researchers can easily alter the design shown in Figure 4 to hold a smaller or larger magnet and a smaller or larger smooth rod.

**Figure 5:**
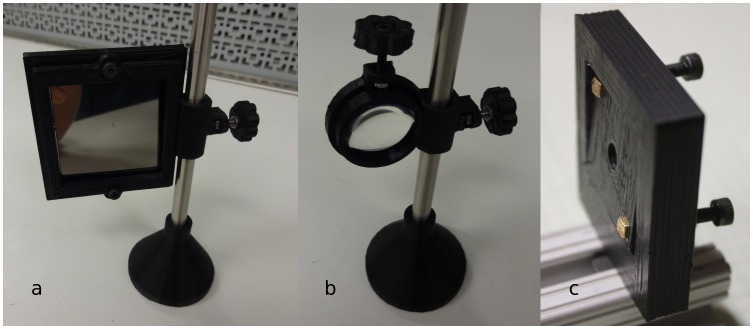
a) Static square filter holder, b) static circular filter holder and c) kinematic mirror or lens holder.

**Figure 6:**
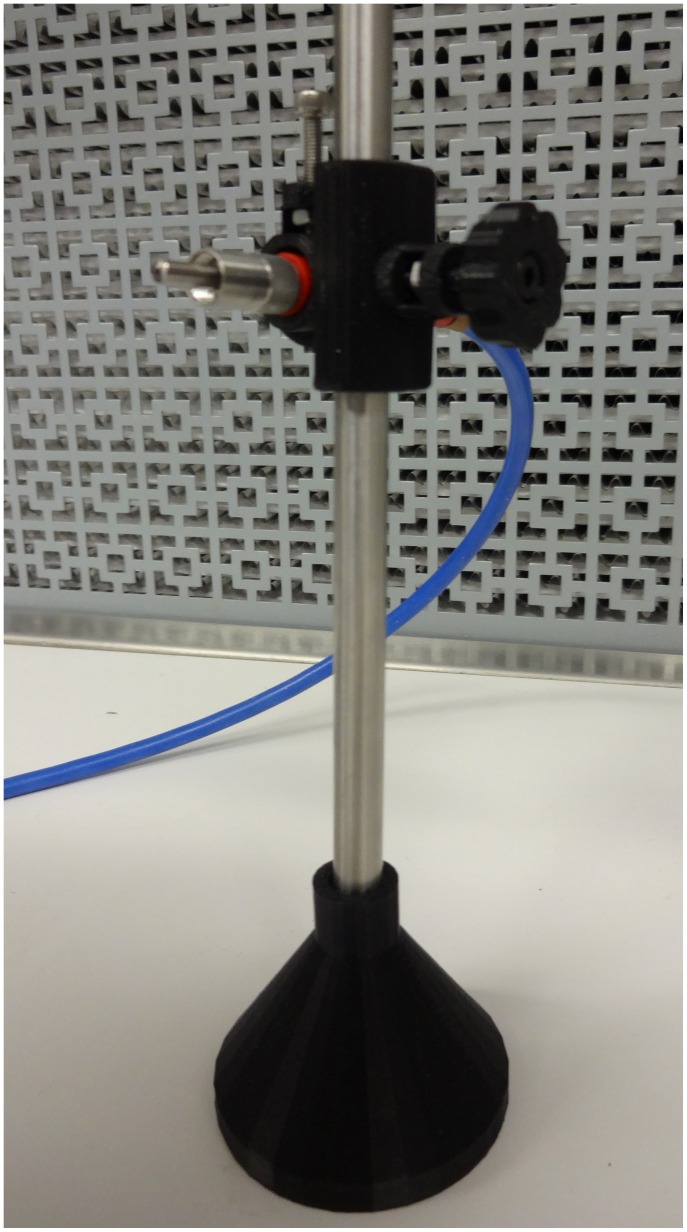
Static fiber-optic holder.

**Figure 7:**
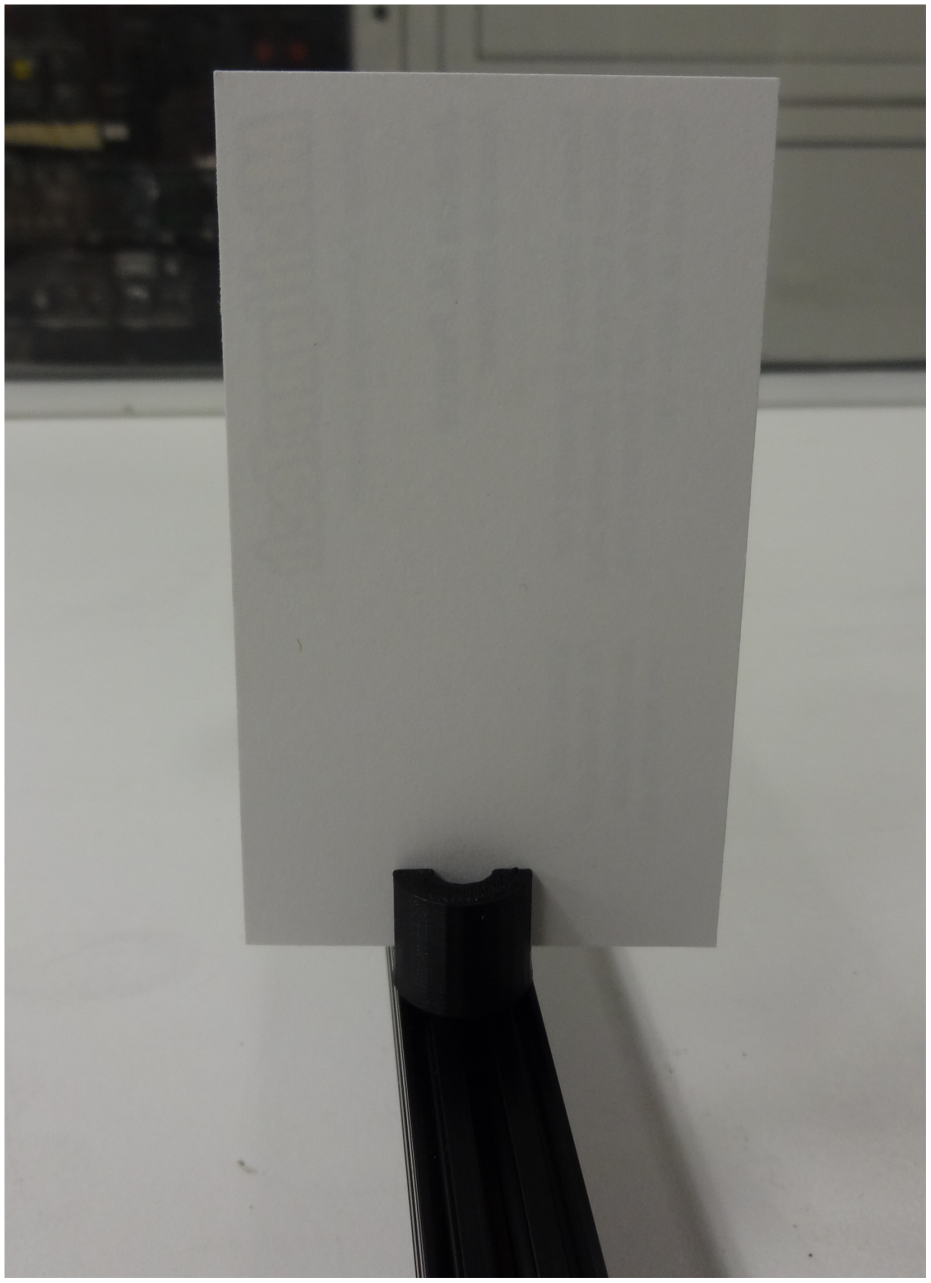
Screen holder.

**Figure 8:**
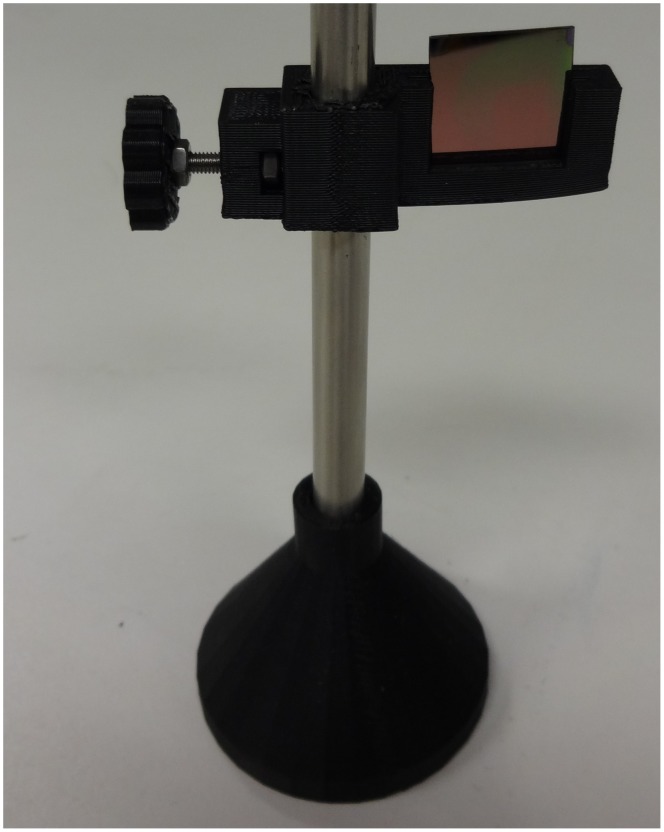
Sample holder.

**Figure 9:**
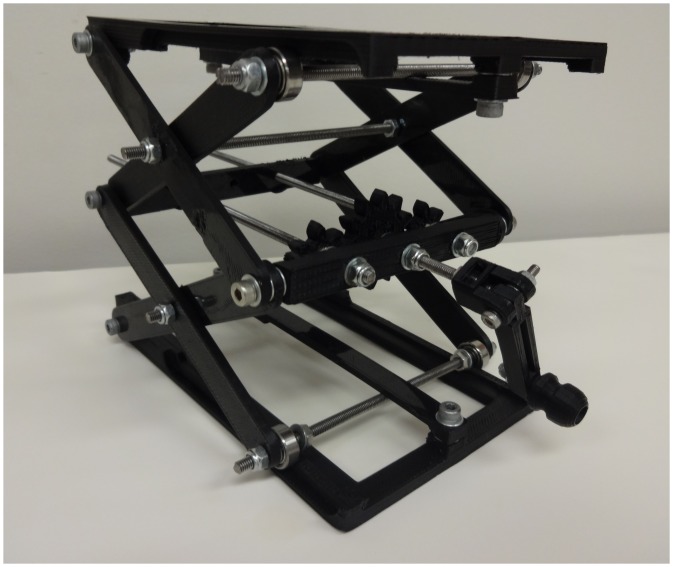
Open-source lab jack.

Numerous components can then be coupled to the 8 mm diameter smooth rods and adjusted in the z-vertical axis to build up an optical assembly including a static filter or lens square (Figure 5a) [36] or circular holder (Figure 5b) [37], kinematic mirror or lens holder (Figure 5c) [38], static fiber-optic holder (Figure 6) [39], screen holder (Figure 7) [40] and sample holder (Figure 8) [41]. The filters in the square holders are fixed in position using m3 screws and nuts and a filter bracket, while for the circular holder a set screw is applied as seen in Figure 5. Both holders can be adjusted for other sizes of filters or lens easily using OpenSCAD. The kinematic mirror or lens mount is partially-parametric design used for steering optics. It contains a living hinge in one corner and two magnets in the adjacent corners, which are attracted to a pair of set screws used to adjust the mirror angle. The static fiber-optic holder was designed to hold a 7.7 mm diameter fiber optic cable. The screen holder is designed to support a screen or card and can be mounted on optical rail with a m3 screw-nut pair. This semiconductor sample holder shown in Figure 8 is designed to hold a semiconductor wafer part on a smooth 8 mm diameter rod. It allows to change samples easily using tweezers with one hand only. More complex, multi-component optics equipment can also be fabricated using this method such as an open source lab jack, which is a height adjustable platform for mounting optomechanical sub-assemblies as seen in Figure 9 [42]. Again, the platforms themselves can easily be adjusted and customized by users to hold any other type of mount.

**Figure 10:**
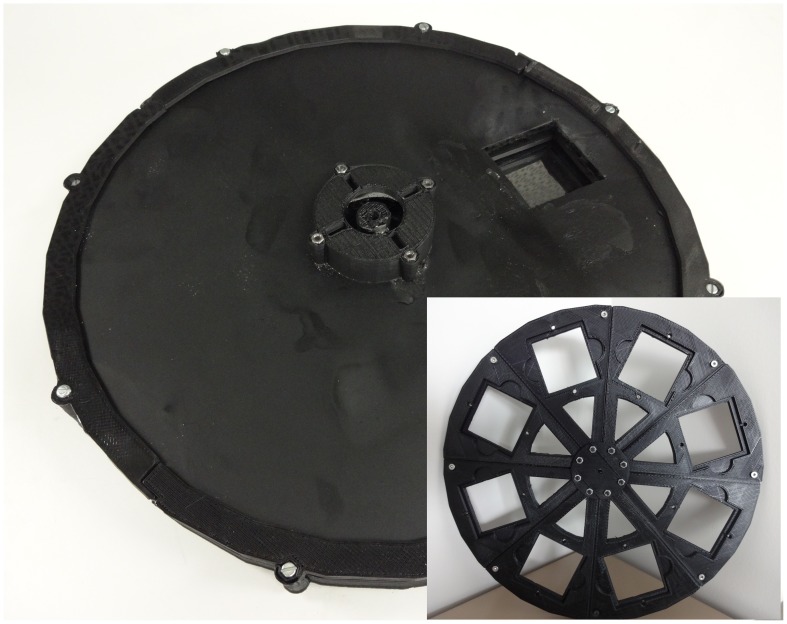
Parametric automated filter wheel changer.

3-D printing and the Arduino can be combined to create automated dynamic optics systems such as the open-source parametric filter wheel shown in Figure 10 [26]. This version of the device has printable wheel with eight filter slots equally spaced by 45 degrees, an Arduino Uno microcontroller, a stepper motor, a discrete optical switch flag, a sensor composed of an output, two ends and a light beam that goes from an end to the other. When the light beam is blocked the output signal changes depending on the logic used (e.g. from high to low). Through a computer interface the user can set the wheel position to the desired filter by clicking on the buttons displayed on the screen, also an indicator provides the current position of the filter wheel. The user’s input is read by the Arduino’s serial communication port, the optical switch flag denotes the filter wheel’s origin (e.g. assuming the filters are indexed from 0–7, the filter 0 is detected when it passes through the sensor). Each time the device is turned on or restarted it rotates until it finds the origin. Using the current location of the filter stored inside the memory and the optical switch flag information as feedback, the Arduino interprets the input and drives the stepper motor following the logic created by the program code until the desired filter is positioned and the input matches the output. The logic always uses the shorter path until the next filter by calculation the difference between the next and the current filter, and always passes through the origin when is possible to maintain calibration.

## Discussion

### Advantages

The OpenSCAD designs and STL files for the entire open-source optics library [26], [31]–[42] were posted on Thingiverse, a digital design repository, which also provides links to all the control software for the Arduino. Any researcher, scientist, or teacher, whether professional or amateur with access to a low-cost 3-D printer can utilize the designs to radically reduce the cost of optical support equipment as summarized in Table 1. As can be seen in Table 1 cost reductions over 95% are common with some components representing only 1% of the current commercial investment. Dozens of small companies offer kits or pre-built open-source 3-D printers, which are easily found on the Internet. With even assembled RepRap printers costing around US$1000 [43], printing a relatively simple optics setup or even a single filter wheel easily recoups the investment. The filament is also available on the web from dozens of suppliers (in various colors and polymer types) and recent work has investigated the use of recycled polymer extruders, which decreases the cost of the material for the components by an order of magnitude [44], [45].

**Table 1:** Material and energy costs associated with open-source optics component fabrication compared to commercial prices and percent savings.

Components	Filament Consumption (g)	ABS Costs(USD)^a^	Electricity Cost(USD)^b^	Total Cost(USD)	Estimated Commercial Price(USD)^c^	Percent Savings(com.-open)/com.
Optical rail	–	–	–	10–12/m	320/m	97
Base on Optical Rail- optical foot (2x)- optical mag (3x)- rod base (4x)	39.52	1.50	0.27	3.08	150–730	>97
Filter holder	8.98	0.34	0.06	0.40	58–80	>99
Lens holder	5.35	0.20	0.04	0.24	20–180	>98
Mirror holder	7.40	0.28	0.05	0.33	18–200	>98
Fiber switcher	10.41	0.40	0.07	0.47	22–138	>97
Screen holder	1.55	0.06	0.01	0.07	18	99
Thumb screw (6x)	7.98	0.30	0.06	1.32	12	89
Sample holder	6.00	0.23	0.04	0.27	18–109	>98
Lab jack	133.20	5.06	0.92	5.98	35–1000	83–99
Automated filter wheel changer	295.1	11.21	2.02	20.43	1000–4250	>98
Optical base (4x)+steel sheet vs. optical table 1 m^2^	46.28	1.76	0.32	25.58	3619–5288	>99

Notes:

a The price of 3 mm ABS filament is $0.038/gram [3D Printer Stuff. Available: http://www.3dprinterstuff.com/shop/page/4?shop_param=Accessed 2012 Oct 19.].

b The national average cost of electricity is 11.53cents/kWh [US Energy Information Administration. Available: http://www.eia.gov/beta/enerdat/#/topic/7?agg=0,1&geo=g&endsec=vg&freq=A&start=2008&end=2011&charted=1 Accessed 2012 Oct 19.] and the electricity cost was derived from multimeter is 0.006925 kWh/gram (using 3 mm ABS) assuming a Prusa RepRap 3-D printer.

c Commercial prices were derived from website data from various vendors including: Edmund Optics, Thorlabs, McMaster-Carr, AutoMate Scientific, and Pasco.

The ability to custom manufacture optics equipment to specifications within a university, government, or industrial laboratory not only ensures that the components are exactly what the research needs, but it also saves time. For example, it is much faster to print a pre-designed component than to go to a lab supply store if there is one in the area or even order online with next-day shipping. The value of timely access to experimental equipment can hardly be overstated for researchers, as delays due to out-of-stock equipment and shipping are well known problems for all researchers doing advanced experimental work. If a critical component is out of stock from suppliers, designing and printing it within a lab can literary save weeks. As the components of the open-source optics library are parametric, customizing the part and having a finished design ready for printing is extremely fast and easy. However, it should be pointed out that time savings are highly component specific. 3-D printing in general is more time consuming on a per part basis than any mass manufacturing process, but there are savings associated with ordering, stocking and shipping for self fabrication. In addition, if a new component of significant complexity needs to be designed from the beginning this can be more time consuming than simply ordering a commercial component. However, if the component needs to be customized, self fabrication again can be much faster.

Although some of these optical components are less precise than commercial versions (as will be discussed in the next section), often experimental setups contain overly engineered components and the open-source tools described in this paper provide more flexibility. For example, eliminating an optics table in exchange for using magnetic bases, a steel plate or re-purposing an existing steel case desk, not only radically reduces the setup costs, but also enables far more flexibility, time-saving and ease of reconfiguring an optical experimental setup.

The open-source optics library provides an easy way to design and conduct educational experiments in a cost-saving form. Particularly, for teachers and lab instructors planning multiple educational optical benches for classes using the open source optics library would provide substantial savings. For example to outfit an undergraduate teaching laboratory with 30 optics setups including 1 m optical tracks, optical lens, adjustable lens holder, ray optical kit, and viewing screen, the total cost would be less than $500 using the open-source optics approach as compared to $15,000 for commercial versions, providing over $14,500 in savings. Thus, using the approach described in this paper the total cost can be reduced by an order of magnitude, allowing access to experimental setups in far more locations (e.g. high schools). Besides economic savings the ability to custom fabricate equipment with tolerable accuracy and precision for elementary, middle and high school education in basic science, chemistry, and physics can save teachers time, similar to the benefits enjoyed by professional researchers. So for example, the rod holder could be easily adapted to the diameter of a birthday candle again significantly reducing the cost of any light source. With the open-source optics library many middle school, high school and college-level experiments can be preformed. The students could be responsible for creating the scales on paper secured directly under the rail, which could also be used to draw and label a ray diagram that includes the positions of the light source, lens and viewing screen. The students learn to describe the image, real or virtual, upright or inverted, larger than object or smaller, gaining knowledge about the properties of the mirrors and lenses. As a useful exercise students within the classes could be responsible for fabricating some of their own optics equipment with 3-D printers, or improving designs; this construction activity cultivates students practical abilities and exposes them to useful engineering skills (e.g. geometry, CAD and additive layer manufacturing) as well as the open-source philosophy. Finally, it should be noted that there are substantial energy and environmental savings made possible by distributed manufacturing [45] and although optics equipment in general does not provide a relatively large environmental burden, it again is useful as a teaching tool for students when considering the design of other more environmentally-destructive types of equipment.

It should also be pointed out that there is a burgeoning collection of 3-D printing services, where users can upload a design and receive a 3-D printed part in the mail. These services can be used for more complex parts, components needing to be printed out of more advanced materials like metals, or for schools/programs interested in trying out the potential of 3-D printing for the science labs before embarking on 3-D printing themselves.

As the RepRap is highly portable and there have already been efforts to construct solar-powered 3-D printers, it can easy to support optics education in rural areas or developing countries and education institutions with funding constraints [10], [46]. It is important to note, the benefit of this approach for international scientists. Experimental science is severely underfunded in most of the developing world as compared to Europe, the U.S. and Japan. At the same time the majority of humanity still lives in these regions and they possess talented scientists, many of whose training and theoretical background rivals the West. Yet these scientists are handicapped by not having access to experimental equipment. This open-source approach would thus help enable more scientists to join the experimental side of the world scientific community, which is presumed to be a benefit for everyone.

### Limitations

There are several limitations of the current open-source optics library. First, it is far from complete. There are hundreds of additional optical components that could be developed using this method that have not been designed yet. At the same time, there are many components that can not be developed with the current technologies discussed here (e.g. lenses). Secondly, there is sometimes a trade-off between precision and cost, both for commercial optical equipment and the open-source variants discussed here. For example, the fundamental properties of the RepRap limit printed part resolution. The 0.5 mm Reprap extruder nozzle has a 2 mm minimum feature size, 0.1 mm positioning accuracy, and layer 0.2 mm thickness. There has already been considerable work done to move to smaller filament diameters and nozzle sizes, but extremely high tolerances are still not accessible to low-cost open-source 3-D printers. In the same way, the Arduino platform is meant primarily for prototyping so microcontrollers designed for a specific purpose can have better performance or have properties not available currently (e.g. able to hand higher voltage ranges). Thirdly, there are no warranties associated with open-source optics equipment – the user gets what they make. Thus the quality of the components and the work that can be done is sometimes user dependent. For example, parts printed from ABS, the same polymer that makes up Lego blocks, is relatively robust if printed with sufficient fill. However, the mechanical strength of the components is dependent on the quality of the print, which will vary among printers/users. Further work is needed to determine if the layered material can endure consistent rough manipulations in educational applications so the lifetimes of printed parts can be compared to industrial-manufactured injection molded components. Finally, although the sharing of open-source optics designs significantly reduces the complexity of replicating equipment there are still substantial knowledge sets necessary to take full advantage of the power offered by the open source approach. These knowledge sets can act as barriers to entry for researchers and educators. For example, the software knowledge necessary to operate OpenSCAD, the printing software, and the Arduino coding is largely dependent on prior exposure to and basic programming ability and skills of the user. However, there are a vast array of free on-line tutorials, videos, examples and instructional materials available for the novice users for all three types of software.

### Impacts and Future Work

This paper has demonstrated an open-source optical library, which significantly reduces the costs associated with much experimental optical equipment while also enabling the production of highly-customizable designs. These reductions in costs and increases in flexibility are likely to boost technical development in any field that utilizes optical testing equipment. At the same time that it assists the research community it can also radically reduce the cost of education, amateur and DIY science [18]. This is thus likely to encourage more DIY science and young people to enter science, technology, engineering and mathematics (STEM) fields [47], [48]. An example of this approach is being preformed by The Public Laboratory for Open Technology and Science (Public Laboratory). The Public Laboratory mission is: “Using inexpensive DIY techniques, we seek to change how people see the world in environmental, social, and political terms. We are activists, educators, technologists, and community organizers interested in new ways to promote action, intervention, and awareness through a participatory research model.” Thus, using inexpensive components and open-source software Public Laboratory has developed an Android-based cellphone spectrometer with a range of 400–900 nm and a resolution of 3 nm [49]. This spectrometer greatly expands the potential users of spectroscopy and can be used to identify dyes in laundry detergent, to test grow lamps, and to analyze food. Members of the Public Laboratory write detailed protocols for each application, making science more a part of everyday life [49].

Future work on the technological development of this open-source optics model is necessary to meet the full potential of the concept. Although 3-D rapid prototyping is currently used primarily in research and development and thus contributes only to a tiny fraction of global manufacturing, the processes has an enormous potential to fabricate more complex components with improved precision and materials selection. RepRap-like printers need to be developed that can print other materials with sufficient resolution to produce lenses, filters, and mirrors. With advanced deposition techniques, chemically active components and optical coatings could be printed. Thus mirrors and filters with different wavelength ranges could be custom digitally fabricated by simply depositing desired species. In addition, 3-D printers in the future are expected to have higher resolutions, which enable other applications. Taken to the atomic limits, 3-D printing can be applied to nanofabrication, nanoimprinting, and nanoscale deposition techniques that open up further applications. With strong cases being made for more open-source approaches in nanotechnology [50], the co-development of these concepts could provide enormously powerful tools. For example, a micro-scale 3-D printer could print the integrated circuit used in open-source optics or a nanoscale 3-D printer can print the gratings used for light diffraction. In addition, 3-D printers and nanotechnology provide the opportunity to fabricate digital designs of chip-like optical systems further depressing costs of experimental equipment and opening the possibility of making them as ubiquitous as cell-phone cameras.

The methodology described here can also be used to make more advanced optical devices such as spectrometers, monochromoters and ellipsometers. Not only optical apparatuses can be built in this way; mass spectrometers, chromatography and even x-ray diffraction systems and other equipment are theoretically printable to a large extent using next-generation open-source 3-D printers. On the other hand, as the 3-D printer itself is reduced in size, it is also possible to have built-in 3-D printers inside the large machines, serving as an in-situ assistant for components replacement, circuit reparation and in-situ design and in-situ fabrication. Symes et al. have already reported the application of 3-D printer as reactionware for chemical synthesis and analysis [51]. This enabled reactions to be initiated by printing the reagents directly into a 3-D reactionware matrix and to be monitored *in situ*. The construction of a relatively cheap, automated and reconfigurable chemical platform makes the techniques from chemical engineering accessible to traditional synthetic laboratories [51].

As discussed previously a large number of open-source software programs and open-source databases have been built in recent years that benefit scientists [4]–[15]. As open-source hardware becomes more mainstream and open-source data sharing allows everyone everywhere at anytime to design, build, share and comment on open-source hardware the utility of the approach will create a virtuous cycle. As people design, build, share and comment, they contribute value to the open-source communities, which everyone again can benefit from and thus encourage more participation. As this paper has demonstrated, this has already started in the field of optics, can spread throughout the rest of the sciences and is applicable to most fields.

### Conclusions

This article introduced a library of open-source 3D-printable optical components to provide an extremely flexible, customizable, low-cost, start of a public-domain library for developing both research and teaching optics hardware. The results show that using this open-source optics method can reduce costs of many optical components by 97% or more. It is clear that this method of scientific hardware development enables a much broader audience to participate in optical experimentation both as teaching and research platforms than previous proprietary methods.
